# Weak genetic divergence and signals of adaptation obscured by high gene flow in an economically important aquaculture species

**DOI:** 10.1186/s12864-025-11259-9

**Published:** 2025-02-05

**Authors:** Bernarda Calla, Jingwei Song, Neil Thompson

**Affiliations:** 1 Pacific Shellfish Research Unit, United States Department of Agriculture, Agricultural Research Service, Newport, OR USA; 2 Coastal Oregon Marine Experimental Station, Oregon State University, Newport, OR USA

**Keywords:** Gene flow, Divergence, Pacific oysters, Adaptation, Captivity, Inbreeding, Diversity

## Abstract

**Background:**

The genetic diversity of a population defines its ability to adapt to episodic and fluctuating environmental changes. For species of agricultural value, available genetic diversity also determines their breeding potential and remains fundamental to the development of practices that maintain health and productivity. In this study, we used whole-genome resequencing to investigate genetic diversity within and between naturalized and captively reared populations of Pacific oysters from the US Pacific coast. The analyses included individuals from preserved samples dating to 1998 and 2004, two contemporary naturalized populations, and one domesticated population.

**Results:**

Despite high overall heterozygosity, there was extremely low but significant genetic divergence between populations, indicative of high gene flow and/or little variability from founding events. The captive population, which was reared for over 25 years was the most genetically distinct population and exhibited reduced nucleotide diversity, attributable to inbreeding. Individuals from populations that were separated both geographically and temporally did not show detectable genetic differences, illustrating the consequences of human intervention in the form translocation of animals between farms, hatcheries and natural settings. Fifty-nine significant F_*ST*_ outlier sites were identified, the majority of which were present in high proportions of the captive population individuals, and which are possibly associated with domestication.

**Conclusion:**

Pacific oysters in the US Pacific coast harbor high genetic heterozygosity which obscures weak population structure. Differences between these Pacific oyster populations could be leveraged for breeding and might be a source of adaptation to new environments.

**Supplementary Information:**

The online version contains supplementary material available at 10.1186/s12864-025-11259-9.

## Background

The genetic diversity of a population determines its ability to adapt to episodic and fluctuating environmental changes. For domesticated species, including those used for agriculture, genetic diversity is a key component of their potential for genetic selection and for adaptation to non-natural environments. Under this view, comprehensive knowledge of the gene pool of a species remains fundamental for developing practices that maintain its health and productivity. This knowledge may also inform establishment of founder populations for broodstock and enable the measuring and utilization of the organism inherent resiliency. In addition, since the genetic diversity of a species is the result of an interplay between gene flow, genetic drift, and selection, it offers a window into its evolutionary and demographic history.

The Pacific oyster *Magallana gigas* (previously *Crassostrea gigas*, Thunberg, 1973), a species native to the Pacific Coast of Asia, was introduced to the Pacific Coast of the United States through multiple independent transplants from Northeast Japan occurring mainly during the period between 1902 and 1960 [1]. These introductions followed the depletion of native oyster (*Ostrea lurida*) populations due to overharvesting and pollution [1, 2]. Because of the inherent fast growth and high reproductive potential of *M. gigas*, this species quickly became established in commercial aquaculture, being now the most cultivated and consumed shellfish species in the U.S. and around the world. Annual production of Pacific oysters in the U.S. reached 2,433 metric tons in 2021 (https://www.fisheries.noaa.gov/). In the 1980s, efforts focused on developing local Pacific oyster hatcheries that could satisfy the demand for seed, eliminating the dependency on imports from Japan and on seed collections from natural settlements occurring in limited areas of the North American Pacific Coast [3]. The Oregon State University Molluscan Broodstock Program (OSU-MBP) was initiated in 1996 using multiple collections of naturalized Pacific oysters from Willapa and Dabob bays (Washington, US) and from Pipestem Inlet, Vancouver Island (British Columbia, Canada). These founders were used to establish a hatchery-domesticated population maintained through artificial spawning and selected for yield traits and, more recently, for field survival in a OsHV-1 positive bay [4–7]

The unique history of the origin, introduction, naturalization, and domestication of *M. gigas* in the United States Pacific Northwest, together with the availability of genetic material from both “museum” specimens (i.e., samples collected and preserved since the 1990s) and contemporary naturalized and captive populations offers an opportunity to evaluate the imprint of environmental challenges and of hatchery and selection practices on the genetic pattern within this species. In-depth knowledge of the available genetic diversity and population structure of the Pacific oyster is also paramount for the understanding of its potential to adapt to existing and future environmental challenges. Because of the repeated introductions that may have resulted in genetic remixing and/or bottlenecks, and due to hatchery and out-planting practices, *M. gigas* genomic variability and population structure on the U.S. Pacific West Coast could be complex [8]. In addition, typical population genetic assumptions may not hold for this species due to its high fecundity, high larval dispersion, and variable reproductive success [9, 10]. *M*. *gigas* is known to harbor high levels of DNA polymorphism and heterozygosity [11], and exhibit “reproductive sweepstakes” which limits effective population size in specific cohorts [10], affects Mendelian segregation ratios via null alleles, and increases genetic load [12, 13].

Several studies have documented genetic variability in *M. gigas* from different regions of the native and introduced species range. Zhang and collaborators [14] assembled a reference genome from a 4th generation inbred *M. gigas* individual and identified over 3 million SNPs by comparing reads from a wild oyster against this newly assembled genome. This study had the obvious limitation that a single re-sequenced individual cannot represent variability between or within populations. More recent studies have used larger numbers of individuals spanning geographical areas, for example, H Qi, K Song, C Li, W Wang, B Li, L Li and G Zhang [15] documented 2.7 million SNPs by resequencing 472 Pacific oyster individuals from China, Japan, Korea, and Canada. A subset of the identified SNPs was used to construct a high-density genotyping array [15]. Variation in *M. gigas* European populations was investigated by sequencing 203 individuals from eight different geographical sources to build a medium-density array that also contained SNPs from *Ostrea edulis* (the European flat oyster); the study identified 12.4 million SNPs [16]. DLJ Vendrami, RD Houston, K Gharbi, L Telesca, AP Gutierrez, H Gurney-Smith, N Hasegawa, P Boudry and JI Hoffman [17] reported high genetic differentiation between southern and northern Pacific oyster populations from Western Europe using a SNP array. Low effects of translocation (movement of juveniles between natural settings and farms) and farm and hatchery practices on genetic connectivity between populations were identified for Canadian Pacific Northwest populations using roughly 17,000 markers identified via RAD-seq [18]. Within the native range, low coverage whole genome resequencing identified 12.2 million SNPs among two wild populations from Northeast Japan and Northeast China and two breeding populations derived from each wild collection [19]. Yet other research has found low genetic diversity and low population differentiation based on over 2 million SNPs in seven wild populations of *M. gigas* in Dalian, China, the major producer of Pacific oysters in the region [20].

To date, no studies have documented genetic variation among populations from the U.S. Pacific Coast using whole genome resequencing. Available studies for this geographical range have been restricted to those utilizing a limited number of markers (e.g., allozymes and low-coverage SNPs markers) [9] or reduced-representation methods (e.g., RAD-seq) [18]. The main factor precluding whole genome scans that involve a high number of samples is cost, but low coverage sequencing when paired with the appropriate analysis tools, can provide a cost-effective way of measuring diversity with larger sample sizes. In the present study, we obtained a catalog of current genetic diversity in *M. gigas* populations along the U.S. Pacific Coast, including naturalized and aquaculture populations, as well as founders and contemporary populations. Our aims were: to evaluate extant genetic diversity, and to identify potential signatures of historical genetic selection resulting from either naturalization to the U.S. Pacific Coast or artificial selection and hatchery practices. For this purpose, we collected 100 total individuals from 5 temporally and geographically segregated populations of *M. gigas,* including individuals that were part of the OSU-MBP program (a founding cohort and a cohort spawned in 2021), as well as contemporary naturalized individuals from Willapa Bay, Washington, and from a presumably self-recruiting population in San Diego Bay, California. These samples come from a range of geographies and time points, providing both: a broad perspective on the genetic diversity range across selective breeding populations and naturalized individuals, and a perspective on the effects of random genetic drift from historical samples to present day samples. In addition, these sampling and analysis provided insight into the potential for aquaculture introgression in a naturalized population.

## Methods

### Sample collection

**Fig. 1:**
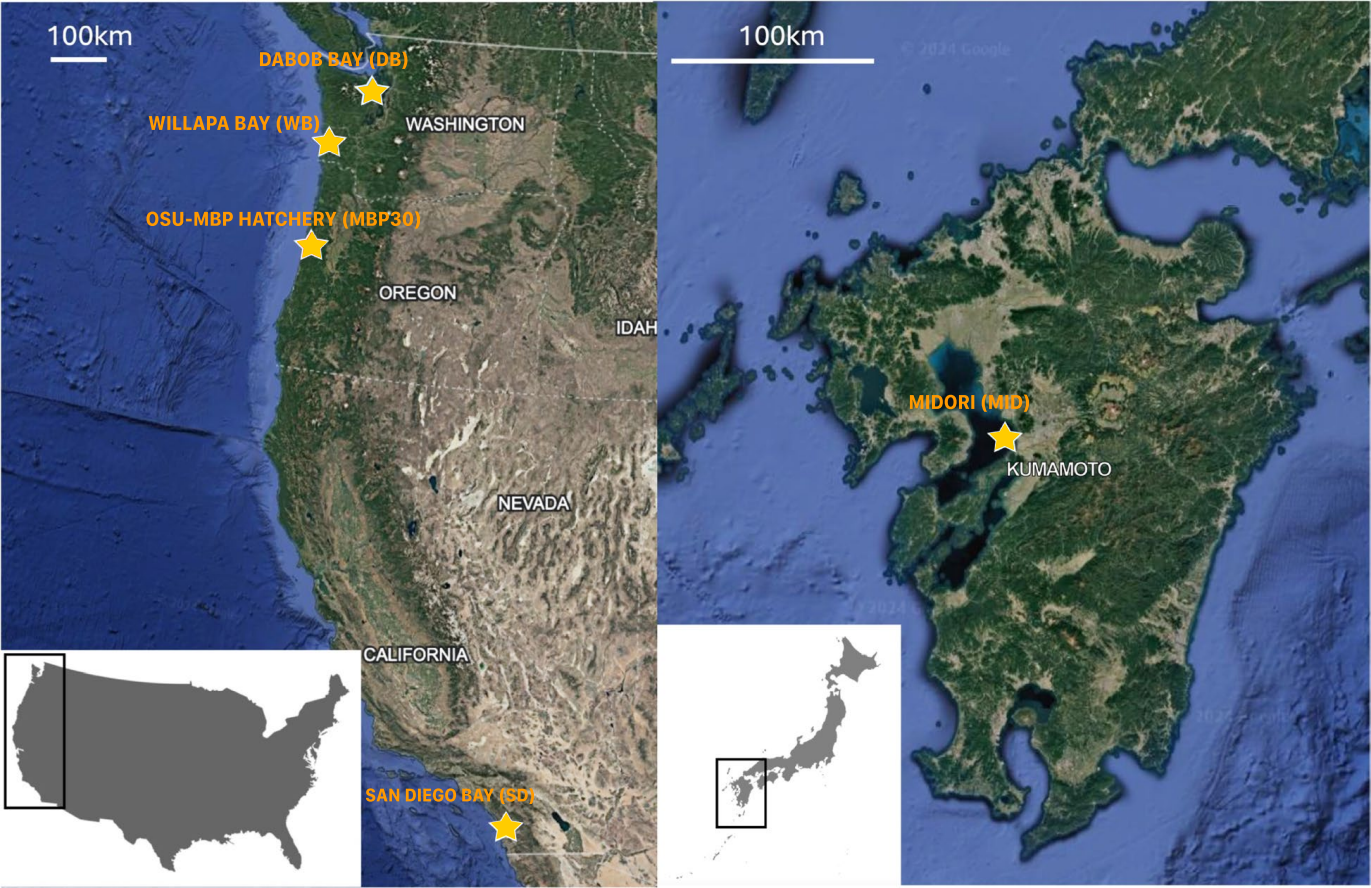
Sampling locations of Pacific oysters (*Magallana gigas*) along the US Pacific Coast. Five populations were sampled, locations are marked with a star. BLUE = Dabob Bay (MBP6 from OSU-MBP); BLACK = Willapa Bay (WB); GREEN = Midori (MID from OSU-MBP); RED = MBP30 (from OSU-MBP); San Diego Bay (SD). OSU-MBP = Oregon State University Molluskan Broodstock Program

Pacific oyster samples from the U.S. Pacific Coast were obtained from five populations (Fig. 1). Adductor or mantle tissue was taken from each animal and preserved in liquid nitrogen or 95% ethanol. U.S. naturalized Pacific oysters were sampled from two sites: Willapa Bay, Washington (WB), and San Diego Bay, California (SD). Animals from the WB population were obtained from an oyster bed (“Parcel A”) near Nahcotta, Washington, in 2022. SD animals were collected from a pier located approximately 125 m south of Tuna Harbor in San Diego Bay; this location experienced oyster mass mortalities due to Ostreid Herpesvirus microvariant (OsHV-1 µvar) outbreaks in 2018 and 2020 [21]. Individuals for this study were collected in July 2020 while the mortality event was occurring, but sampled animals were not exhibiting symptoms of infection or stress. These samples represent extant naturalized Pacific oyster populations for which comparison to aquaculture populations are made.

Two other populations were obtained from OSU-MBP. MBP cohort 6 (MBP6) samples were obtained from a collection of preserved individuals dating to 1998; these oysters were naturalized animals collected from Dabob Bay in 1996 and used as broodstock to initiate, in part, the OSU-MBP selective breeding program [6]. The MBP cohort 30 (MBP30) samples were juveniles produced in 2021, these animals represent the seventh generation of the MBP and have a mixed lineage (founders were from different locations, and descendant families were widely intercrossed) [4, 6]. The MBP30 animals were randomly chosen from all crosses made in cohort 30, with 1 oyster per family being sampled. No full siblings are present in the sequenced MBP30 samples. Limited shared recent ancestry exists within the MBP30 samples, with 7 pairs of MBP30 samples having a first-cousin relationship. The kinship coefficient of MBP cohort 30 sample pairs ranges from 0.032 to 0.178, with a mean kinship coefficient of 0.107, slightly less than a half-sibling relationship. Both MBP cohorts belong to the “Miyagi” population, which was repeatedly introduced to the U.S. from Northeast Japan starting in the 1920s [1]. A fifth population (MID) was obtained from preserved individuals that were collected in 2004 from the Kumamoto Prefecture in Southern Japan by the OSU-MBP. The samples were taken from originally collected individuals (G0), which were later spawned to create the “Midori” population [22]. This population (MID) has undergone little to no selection and is currently cultured widely along the U.S. Pacific coast.

### Whole genome resequencing

DNA extraction, library construction, and sequencing were carried out by the OSU Center for Quantitative Life Sciences. Libraries were constructed using the PrepX DNA library prep kit (Takara) and sequenced in an Illumina NexSeq 2000 with a P3 flow cell to obtain 150 bp paired reads. The target read coverage was approximately 5.6X per sample.

### Variant calling

The resulting sequencing reads were checked for quality with FASTQC v.0.11.9 [23]; reads with trimmed adapters were then mapped to the reference *Magallana gigas* genome (NCBI GenBank # GCA_902806645.1) [24] using BWA v. 0.7.17-r1188 with the *mem* algorithm. For compatibility with the downstream pipeline, the BAM had read groups (RG) added with the ‘AddOrReplaceReadGroups’ from Picard v. 2.27.1 (https://broadinstitute.github.io/picard/). Mapping quality and coverage depth were evaluated with Qualimap v. 2.3 [25].

The following steps were completed using the tools and Best Practices workflow from GATK v.4.1.4.1 [26, 27]. Duplicates were marked with MarkDuplicatesSpark from, the GATK Spark implementation of Picard’s MarkDuplicates; this process also sorted the records by coordinate. Next, SNPs and indels were called with HaplotypeCaller, generating GCVF files for each sample. All GVCFs were then merged into a database with GenomicsDBImport first without and then with the “all-sites” option to maintain both variants and invariant sites. Joint genotyping was done with GenotypeGVCFs using the database generated in the previous step as input. For memory efficiency when using the “all-sites” flag, both GenomicsDBImport and joint genotyping were run on intervals created by splitting the reference genome into 18 segments. All files with called genotypes were then merged into ten separate chromosome files plus one file containing the 236 unmapped scaffolds for remaining steps. Best filtering strategies were explored in a subsample of the called genotypes using vcftools [28] and ggplot package in R v.4.3.1. Filtering was done in two separate steps following GATK best practices recommendations. First, a hard filtering step was applied using the following cutoffs: Fisher Strand > 60.0; Strand Odds Ratio (SOR) > 3; RMS Mapping Quality (MQ) < 40; Mapping Quality Rank Sum Test (MQRankSum) < -4.0; Mapping Quality Rank Sum (MQRankSum) > 12; Quality by Depth (QD) < 2.0; Read Position Rank Sum (ReadPosRankSum) < -3.0; and combined depth across all samples (INFO/DP) > 500. A second filtering was applied to remove individual genotypes with poor values (based on by-sample fields in the vcf file) with Read Depth (FMT/DP) < 3 and Genotype Quality likelihood (FMT/GQ) < 20 and genotypes which, after filtering off sites in the previous step, had all remaining sites missing. In addition, SNPs within ten bp of indels and with more than two alleles were removed (--SnpGap 10; -m2 -M2). The file containing invariants was filtered separately to preserve invariant and variant sites as input to Pixy software.

To test deviation from HWE, we used the exact test as defined in JE Wigginton, DJ Cutler and GR Abecasis [29] and implemented in vcftools v0.1.17 “--hardy”. The max-missing flag filter was set to no more than 10% missing data. *P*-values were adjusted based on false discovery rate (FDR) [30]. SNP loci with an FDR < 0.1 were considered significant.

### Genetic diversity and population structure

To evaluate genetic structure and relatedness between the populations, linked SNPs were first removed using PLINK2 [31] using -indep-pairswise 50 5 0.5. The resulting pruned set was used in a Principal Component Analysis (PCA). To assess the proportion of ancestry and relatedness from each population, we used ADMIXTURE v. 1.3.0 [32, 33] with the PLINK pruned output; the optimal K-value was evaluated with the ADMIXTURE cross-validation feature with K ranging from 1 to 7. Average nucleotide diversity within populations (π) and average divergence between populations (*dxy*) was estimated with Pixy v.2.1.7 beta [28] using 10 kb non-overlapping sliding windows on the vcf file that had both variant and invariant sites. Per-population π and pairwise *dxy* averages were calculated by adding the raw by-window counts of pairwise differences between genotypes and then dividing by the sum of total raw by-window non-missing sites.

Unbiased Weir and Cockerham F_*ST*_ [34] and Nei’s genetic distances [35] between populations were estimated with StAMMP v. 1.6.3 [36]. The significance of the F_*ST*_ values was calculated with 95% confidence intervals over 100 bootstrap replications. An Analysis of Molecular Variance (AMOVA) was run with 1000 permutations to estimate variance partitioning with StAMMP.

### Outlier detection

F_*ST*_ outliers were analyzed with Outflank v 0.2 [37] using SNPs that were present in at least 80% of the samples. To infer the χ^2^ square distribution against which the outliers were tested, the tails of the F_*ST*_ distribution were trimmed with the Outflank default trimming values but also removing low heterozygosity loci (He values > 0.1). Outliers were detected based on the obtained distribution with a cutoff q-value < 0.01. The detected outliers were mapped back to the chromosomes in the reference genome assembly and were functionally annotated with SNPEff v. 5.1d [38]. Annotations were manually verified for accuracy by querying the NCBI databases.

## Results

One hundred individuals from five populations (WB, SD, MID, MBP6, and MB30) were sequenced (*n* = 20 per population) (Fig. 1). The 2.5 million mapped reads resulted in a mean sample coverage of 5X that was consistent across populations. The GC content of mapped reads was 34.93%, and the mean mapping quality per sample (BAM QC) was 36.76.

### Variants call

The initial SNP calling by GATK contained over 100 million variants, including indels and SNPs, after site-level filtering, sample-level filtering, removing indels, and removing of repetitive regions, there were a total of 57.9 million biallelic SNPs, of which 20.3 million had a MAF > 1% and were not singletons (SNP density 31.3 SNPs per kb of the genome). Only 230 loci deviated significantly from HWE in all five sampling locations. No loci were removed based on the HWE test.

**Fig. 2:**
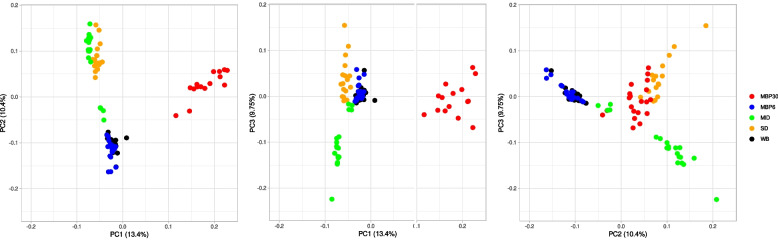
Principal Component Analysis (PCA) based on 20.3 million SNPs across five Pacific oyster (*Magallana gigas*) populations from the U.S. Pacific coast. MID = Midori, SD = San Diego, WB = Willpa Bay; MBP6 = Molluskan Broodstock Program founder from Dabob Bay; MBP30 = Molluskan Broodstock Program Cohort 30

Principal component analysis using the filtered SNPs set after pruning linked sites identified three principal components explaining 33.5% of the total variation (PC1, PC2, and PC3). PC1 explained 13.4% of the variation, whereas PC2 and PC3 explained an additional 10.4% and 9.75% of the variation, respectively. PC1 and PC2 both captured the variation between MBP30 and all other populations, these two PC also showed high overlap between MID and SD populations and showed that MBP6 and WB populations were indistinguishable in the PC space. PC3 separated MID from SD more distinctly (Fig. 2).

**Fig. 3:**
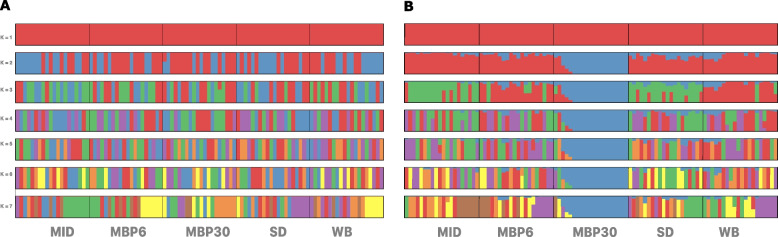
Admixture analysis in one hundred individuals from five populations of Pacific oysters (*Magallana gigas*). Panel **A** Population structure detected using a subsample of SNPs before filtering for rare variants; Panel **B** After MAF filtering showing no population structure. SNPs were called for five populations: MID = Midori, SD = San Diego, WB = Willpa Bay; MBP6 = Molluskan Broodstock Program founder from Dabob Bay; MBP30 = Molluskan Broodstock Program Cohort 30

An ADMIXTURE analysis showed no population substructure: the lowest cross-validation error was found at K = 1 (Fig. 3A). However, a result more congruent with the PCA was seen when using a subsample of 10% of the raw (unfiltered) SNPs, the Q matrix from ADMIXTURE did show that MBP30 could be distinguishable from the other populations, while MID and the SD populations may share a genetic history that separate them slightly from the rest of the populations (with k = 3) (Fig. 3B), indicating that weak population structure signals might effectively get lost when removing out rare variants through MAF filtering [39].

### Genetic variation and differentiation

**Fig. 4:**
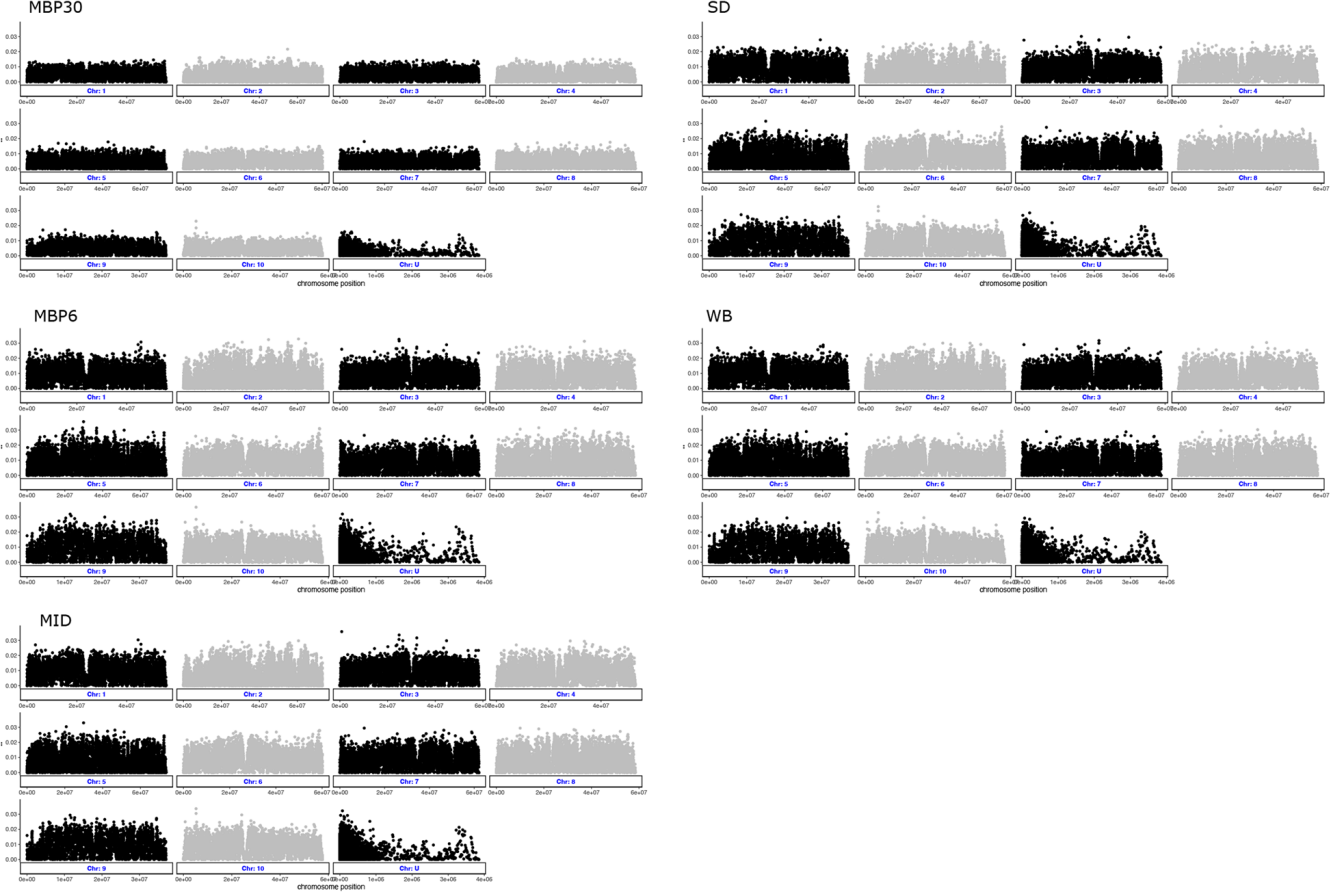
Nucleotide diversity (π) in five populations of Pacific oyster (*Magallana gigas*) from the U.S. Pacific coast. MID = Midori, SD = San Diego, WB = Willpa Bay; MBP6 = Molluskan Broodstock Program founder from Dabob Bay; MBP30 = Molluskan Broodstock Program Cohort 30

To further characterize genetic diversity in the samples, we calculated weighted, unbiased π values across the genome in 10 kb windows using the dataset containing both variant and invariant sites (--allsites output from GATK). MBP30 had the lowest within-population nucleotide diversity (π) equal to 0.0043 compared to MBP6 = 0.0079; MID = 0.00786; SD = 0.00738; and WB = 0.00779. This difference was consistent across the whole genome (Fig. 4).

Population differentiation, as measured using overall pairwise Weir & Cockerham’s *F*_*ST,*_ showed low divergence between populations. MBP30 has the highest divergence with *F*_*ST*_ values between 0.0103 (with MBP6) and 0.0145 (with MID) (Table 1, lower triangle). The lowest F_*ST*_ values were found between MBP6 and WB (0.004). All *F*_*ST*_ values in all the pairwise comparisons were highly significant, with *p*-values < 0.001 obtained by sampling with replacement (100x) and correcting for multiple testing with the Bonferroni method. In general, divergence (F_*ST*_) closely resembled the PCA results. *Dxy* was also used to compare population differentiation, the results showed, again, that MBP30 has the highest divergence with all the other populations. However, pairwise *dxy* values across all the pairwise comparisons were higher and less variable than F_*ST*_ (*dxy* values ranging between 0.07717 and 0.09627; Table 1 upper triangle), indicating that the low within-population nucleotide diversity in MBP30 relative to that of other populations might be a major driver for the divergence results. An AMOVA, to test for partitioning of the genetic variation, found significant differentiation between populations but not within any of the populations (Table 2).

**Table 1:** Measures of genetic divergence. Lower triangle Weir & Cockerham’s F_*ST*_. Upper triangle *dxy*

	**SD**	**WB**	**MBP30**	**MBP6**	**MID**
**SD**		0.080359	0.096269	0.079779	0.080080
**WB**	0.003257		0.091172	0.078181	0.077920
**MBP30**	0.011596	0.009732		0.090906	0.089730
**MBP6**	0.003245	0.000396	0.010290		0.077172
**MID**	0.002795	0.004311	0.014500	0.005008

**Table 2:** AMOVA

	**SSD**	**MSD**	**df**	**sigma2**	***P*.value**
Between populations	0.00360666	0.0009	4	1.84E-05	0
Within populations	0.05077457	0.0005	95	5.34E-04	0.1
Total	0.05438122	0.0005	99		

### Outlier analyses

**Fig. 5:**
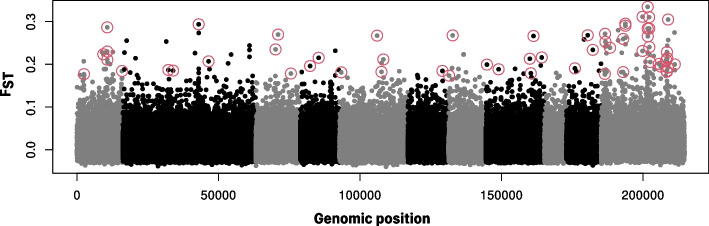
F_*ST*_ values across the genome in five populations of Pacific oysters (*Magallana gigas*). Fifty nine outliers, indicative of local adaptation are marked with red circles. Grey and black color show chromosome delimitation

**Fig. 6:**
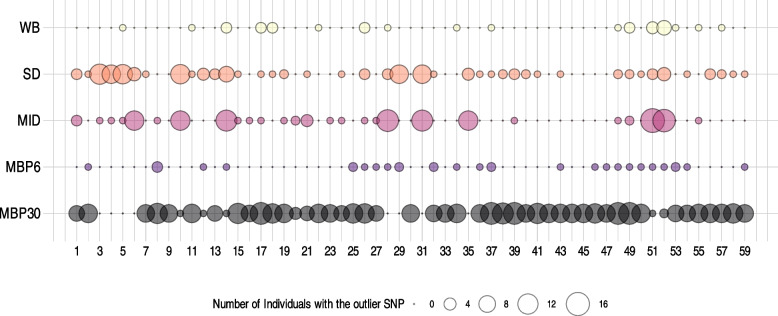
Presence of F_*ST*_ outliers across five populations of Pacific oysters (*Magallana gigas*). Twenty individuals were sequenced per population. Outlier SNPs are those that did not fit in the right-tailed χ^2^ distribution of F_*ST*_ calculated across the whole genome (q-value < 0.01). Vertical bars are the 59 F_*ST*_ outlier sites in order of genomic position. Bubble size indicates the number of individuals that had the alternative allele(s) either as homozygous alternative allele or as heterozygous

The Outflank software, which conservatively fits the distribution of F_*ST*_ from neutral loci to a χ^2^ square distribution, identified 59 top outlier candidate SNPs. These outliers were mapped in the reference genome and found to be distributed across nine of the ten contiguous chromosomes and in seven out of the 236 non-assembled scaffolds (Fig. 5). MB30 had the most individuals with outlier SNPs as heterozygous or homozygous alternative allele (51 out of 59), followed by SD (41 out of 59), whereas MBP6 and WB had the fewest SNPs as heterozygous or homozygous alternative alleles (Fig. 6; Additional file 1).

A functional annotation analysis of the genomic regions where the top outliers were found shows that 39% of these sites are in transcribed regions (protein and non-protein coding), and the remaining (61%) are in non-coding regions. SNPs that translate into non-synonymous variants within protein-coding sequences, which could be considered as the most consequential, include two missense variants, one in a transcript coding for “tripartite motif-containing protein 2” (XM_034452193.1) in Chromosome 10 (only present in individuals from the MBP30 population), and another in a transcript coding for “histone-lysine N-methyltransferase SETMAR-like” (XM_034451927.1) in Chromosome 8 (present in more than half of MBP30 individuals and only in up to 3 individuals from each of the other populations). Additionally, eleven outliers (18% of the total) mapped to five long-non-coding RNA (lncRNA) loci (Table 3). Annotation of outlier also included genomic regions that are within 5Kb of the outlier if they did not fall directly in a coding region. Those were classified as “upstream” or “downstream” gene variants following SnpEff annotation scheme. Half of the outliers that did not map into a coding region were within those categories (Table 3).

**Table 3:** Position and functional annotation of outlier sites detected by Outflank

**Outlier number**	**Chromosome**	**Position**	**Variant type**	**Actual or nearest coding sequence (NCBI refseq ID)**	**Sequence type**	**Functional annotation**
1	Chr: 1	7680836	synonymous_variant	XM_034445312.1	transcript	homeobox protein 6
2	Chr: 1	33625290	downstream_gene_variant	XM_011422575.2	transcript	tubulin-specific chaperone C-like
3	Chr: 1	37904677	intragenic_variant	XR_004595737.1	non-protein coding	lncRNA
4	Chr: 1	37904740	intragenic_variant	XR_004595737.1	non-protein coding	lncRNA
5	Chr: 1	37904809	intragenic_variant	XR_004595737.1	non-protein coding	lncRNA
6	Chr: 1	55548026	intergenic_variant>	LOC105335559	transcript	HCLS1-binding protein 3
7	Chr: 2	33394675	upstream_gene_variant	XR_004601088.1	transcript	uncharacterized LOC117688662
8	Chr: 2>	39522736	downstream_gene_variantp>	XM_034466364.1	transcript	epidermal growth factor-like domains
9	Chr: 2	51858408>	intergenic_variant			
10	Chr: 2	59631568	intron_variant	XM_034465519.1	transcript	uncharacterized protein
11	Chr: 3	27743234	intron_variant	LOC105329645	transcript	sentrin-specific protease 1
12	Chr: 3	30213493	intergenic_variant>			
13	Chr: 3	47602303	downstream_gene_variant	XR_004601319.1	transcript	
14	Chr: 4	12185248	intergenic_variant			
15	Chr: 4	22791392	intron_variant	LOC15343840	transcript	growth hormone-regulated TBC protein 1-A
16	Chr: 5	263654	intron_variant	XM_034479567.1	transcript	platelet glycoprotein Ib alpha chain
17	Chr: 5	39465652	downstream_gene_variant	XM_034475582.1	transcript	uncharacterized protein
18	Chr: 5	41465770	upstream_gene_variant	XM_011438014.3	transcript	cell division cycle 5-like protein
19	Chr: 5	41912112	intron_variant	XR_010714176.1	non-protein coding	lncRNA
20	Chr: 6	49280926	upstream_gene_variant	XM_011427417.3	transcript	E3 ubiquitin-protein ligase CHFR
21	Chr: 7	30647	intergenic_variant			
22	Chr: 7	6307936	intron_variant	LOC105329705	transcript	uncharacterized protein
23	Chr: 8	120446	intergenic_variant			
24	Chr: 8	3525666	intron_variant	XM_034452193.1	transcript	tripartite motif-containing protein 3-like
25	Chr: 8	38861094	downstream_gene_variant	XM_034448703.1	transcript	uncharacterized protein
26	Chr: 8	40231691	missense_variant	XM_034451927.1	transcript	histone-lysine N-methyltransferase SETMAR-like
27	Chr: 8	46762427	downstream_gene_variant	XR_002201338.2	transcript	
28	Chr: 8	52522525	upstream_gene_variant	XM_034449879	transcript	uncharacterized protein
29	Chr: 10	20830318	upstream_gene_variant	XM_034455499.1-1	transcript	protein FAM133A-like
30	Chr: 10	33311896	missense_variant	XM_011451359.3	transcript	tripartite motif-containing protein 2
31	Chr: 10	41514923	upstream_gene_variant	XM_034455021.1	transcript	opine dehydrogenase
32	NW_022994786.1	28721	intergenic_variant			
33	NW_022994786.1	35777	intergenic_variant			
34	NW_022994786.1	62891	downstream_gene_variant	XM_011420943.3	transcript	uncharacterized LOC105322303
35	NW_022994801.1	28046	intergenic_variant			
36	NW_022994829.1	71361	downstream_gene_variant	XR_004599051.1	transcript	lncRNA
37	NW_022994829.1	80271	intron_variant	XR_004599053.1	transcript	lncRNA
38	NW_022994829.1	124001	intron_variant	XM_034459954.1	transcript	uncharacterized protein LOC105341878
39	NW_022994829.1	124008	intron_variant	XM_034459954.1	transcript	uncharacterized protein LOC105341878
40	NW_022994852.1	173056	downstream_gene_variant	XM_034460498.1	transcript	uncharacterized protein LOC117685913
41	NW_022994852.1	173152	downstream_gene_variant	XM_034460498.1	transcript	uncharacterized protein LOC117685913
42	NW_022994852.1	303488	intragenic_variant	XR_004599282.1	non-protein coding	lncRNA
43	NW_022994852.1	303501	intragenic_variant	XR_004599282.1	non-protein coding	lncRNA
44	NW_022994852.1	304348	intragenic_variant	XR_004599282.1	non-protein coding	lncRNA
45	NW_022994852.1	313977	intergenic_variant			
46	NW_022994852.1	314413	intergenic_variant			
47	NW_022994852.1	319933	intergenic_variant			
48	NW_022994852.1	328132	intergenic_variant			
49	NW_022994864.1	196193	intergenic_variant			
50	NW_022994865.1	3222987	intergenic_variant			
51	NW_022994890.1	32136	upstream_gene_variant	XM_034461254.1	transcript	zinc finger protein 862-like isoform X1
52	NW_022994890.1	37030	intergenic_variant			zinc finger protein 862-like isoform X1
53	NW_022994890.1	42354	intergenic_variant			zinc finger protein 862-like isoform X1
54	NW_022994890.1	42663	intergenic_variant			zinc finger protein 862-like isoform X1
55	NW_022994890.1	73226	upstream_gene_variant	LOC105348138	transcript	uncharacterized protein LOC105348138
56	NW_022994890.1	122131	intron_variant	LOC117686395	transcript	
57	NW_022994890.1	127673	intragenic_variant	XR_004599579.1	non-protein coding	lncRNA
58	NW_022994890.1	127710	intragenic_variant	XR_004599579.1	non-protein coding	lncRNA
59	NW_022994940.1	16981	intron_variant	LOC105324010	transcript	uncharacterized protein LOC105324010

## Discussion

*Magallana gigas*, like other bivalves, is known to have extremely high levels of genetic polymorphisms. While this variability could be a source of adaptability and a source for genetic selection, it cannot be easily characterized since it is strongly affected by a combination of life history traits (e.g., high fecundity, high larval dispersal, variable sex ratios, variable reproductive success) and the unique demographic history of this species (i.e., *M. gigas* has been artificially transported, distributed, domesticated, and naturalized across the world).

In the present study, we set to investigate the genetic diversity that exists in *M. gigas* populations on the US Pacific Coast to understand the demographic history, the potential for genetic selection, and the effects of naturalization and domestication on this economically and ecologically important species. We detected high heterozygosity through the identification of roughly 20 M SNPs by resequencing one hundred individuals from five different populations from the U.S. Pacific Coast. This is the highest number of variant sites in this species recorded to date, with the second highest from a study that identified 12.2 M SNPs by evaluating populations from a selective breeding program in China with two wild endemic populations from Japan and China [19]. The study by Hu et al., [19] is among the few that also used whole genomic resequencing, while previous studies might have been influenced by the sequencingmethod (e.g. RAD-seq vs. WGS), the number of sites evaluated, the data processing/filtering protocols or a combination of all of the above.

Although we found a high number of single-site variations, the divergence between populations was low. The 2021 cohort of selectively bred animals (MBP30) was the most divergent, whereas the other populations, except for WB and MBP6, could also be distinguished based on their genetic makeup but with overall signals of weak population divisions. These results were supported across multiple tests: PCA showed population subdivisions while ADMIXTURE showed weak structure that appears to get lost when stringently filtering out rare alleles. Independent estimators of divergence (F_*ST*_ and *dxy*) also indicated low but significant differentiation between populations.

By-population F_*ST*_ showed weak but very significant differentiation (0.00973–0.01450, *P* < 0.0001) between MBP30 and the other four populations. These F_*ST*_ values are comparable to those calculated by Sun et al. [9] between a population of *M. gigas* from Hiroshima (Japan) and six from North America (F_*ST*_ = 0.0151–0.0212), and slightly lower than values from a study using 33 loci where multiple MBP cohorts clustered distinctly away from naturalized Pacific coast populations (F_*ST*_ = 0.0218) [40]. Likewise, a study involving a hatchery population of mixed lineages from US, British Columbia, and South America, showed high divergence from local naturalized and other hatchery populations [41]. However, divergence estimates in the latter study were much larger than those obtained in the present work (average F_*ST*_ = 0.06). In another study [20], the authors obtained F_*ST*_ estimates comparable to those in our study when comparing populations in the Dalian Sea, a prominent aquaculture region in China. MBP30 has been reared in captivity and with controlled reproduction for over 25 years, and while there is no record of intentional new introductions of animals to minimize inbreeding during those years, the founding broodstock used to initiate the MBP has been mixed with broodstock from other localities and undergone multiple mating schemes [6]. MBP30 also has the lowest nucleotide diversity (average and by window or chromosome), possibly as a result of inbreeding and/or a reduction in effective population size since the number of crosses per cohort ranged from 24–85 [6]. This result is not unexpected given the domestication history of MBP30 and the challenges in maintaining high genetic diversity and low rates of inbreeding accumulation in aquatic animal breeding programs.

In this study, we used the Cockerham and Weir F_*ST,*_ which (as all other F_*ST*_ statistics) is a relative measure of population divergence largely depending on current within-population diversity (π). *Dxy*, conversely, is independent of extant population diversity and better reflects the relationships between populations that are shared by ancestry [42]. *Dxy* generally supported the F_*ST*_ results, but the values were more similar across pairs of populations involving MBP30, indicating that the lower nucleotide diversity within MBP30 is what causes, at least in part, the elevated F_*ST*_ values in pairs including MBP30. An AMOVA, based on Nei’s genetic distances, also supported significant genetic variability between populations, although AMOVA does not allow for dissecting the specific pairs that diverge, based on all other results, MBP30 is likely the major driver of such variability.

None of the tests we performed detected differences between MBP6 (the broodstock collected from Dabob Bay in 1998) and WB, the contemporary, naturally recruiting population from Willapa Bay collected in 2022. This pair of populations had the lowest F_*ST*_ (0.000396, *p*-value < 0.0001) and overlapped significantly in the PCA. This is an unexpected result since these two populations are separated geographically and temporally, underscoring the challenge in characterizing population genetics in this species. If we assume two generations per year and that the standardized temporal variation (Fs) [43] is expected to be double the F_*ST*_ between two populations [9], temporal change between these two populations is still very low (Fs 0.0096). Although Dabob Bay and Willapa Bay are geographically segregated, there was and continues to be extensive human-led movement of animals between those two locations. These results contrast with the study by Sun and Hedgecock [9] in which large genetic divergence in time for populations collected in Dabob Bay in 1985, 1996, and 2006 was detected. A more direct comparison using contemporary samples from Dabob Bay would be useful to clarify discrepancies.

The SD population, assumed to be a naturalized, self-recruiting population sampled from San Diego Bay, CA, tightly clustered with the MID population, which, in turn, has been in captivity as part of the OSU-MBP in Newport, Oregon, since its collection in Japan in 2004. ADMIXTURE analysis using all SNPs (before filtering for INDELS and rare alleles) supports that these two populations share some weak genetic ancestry. This finding was unexpected, and one possible explanation is that animals from the same region in Japan from where MID came from were somehow moved to San Diego Bay. Unintentional transport via ship ballast and fouling is plausible since San Diego is one of the most active ports on the U.S. West Coast. The data suggests that the progenitors of SD were more likely from Southern Japan near Kumamoto rather than from the Miyagi prefecture in Northeastern Japan, where the bulk of seed imports originated from in the previous century [1]. In addition, after collecting samples for this study, the fact that the SD population is self-recruiting has been questioned. There is at least one report of Pacific oysters established in southern California, although it was unclear, at the time of that report, if these clusters of established oysters would persist since many intentional attempts to introduce Pacific oysters in that region had failed in the past [44]. Recently, an in-depth report of feral *M. gigas* presence on the U.S. Pacific Coast showed that this species has increased in abundance in Southern California and pointed to possible permanent establishment and dispersal [45]. An investigation into the self-recruiting ability of animals from San Diego Bay is warranted but outside of the scope of this study. Importantly, given the population genetic characteristics of SD found here (i.e., low but marked genetic divergence from other populations), this location could be a source of genetic diversity exploitable for breeding purposes.

To evaluate F_*ST*_ outliers, we used the Outflank algorithm developed by Whitlock and Lotterhos [37] based on the first formal method for detecting F_*ST*_ outliers [46]. Importantly, Outflank does not assume that populations are independent, allowing for population pairs to have exchange of migrants and shared evolutionary histories, which we deemed appropriate in this study. MBP30 had the most individuals with identified outlier sites followed by SD, while some outliers were present only in some populations and not in others. Several outliers fell into coding regions and regulatory regions. Importantly, many of the outliers were found in genes encoding lncRNAs, important regulators of transcription through a variety of mechanisms and believed to be involved in immunity and stress response in bivalves [47, 48]. LncRNAs were associated with growth regulation in Pacific oysters [49]. These associations are plausible given MBP’s history of selection for yield (a composite of growth and survival) and more recently for field survival in OsHV-1 positive bay. The biological characteristics among populations varied considerably. The MBP30 samples are the result of captive breeding since the late 1990s and outliers identified within them could indicate domestication loci, or those associated with selective breeding for yield (a combination of meat weight and survival) and/or survival in an OsHV-1 positive environment (Tomales Bay California). The SD naturalized oysters were sampled during an active OsHV-1 microvariant outbreak, but did not show any symptoms of infection. Thus, it’s possible these would have been outbreak survivors, and if simple genetic architecture underlies OsHV-1 microvariant survival then the identified outliers could signify chromosomal regions of effect. The Midori population has an exposure history to OsHV-1 (De Melo 2021) and may also harbor OsHV-1 associated resistance loci that could be detected as outliers in comparison to other populations.

Although we were able to functionally annotate the loci where the F_*ST*_ outliers were identified, both the genome assembly reference used in this study and its associated annotations are still relatively unrefined. Many of the identified outlier sites are in or nearby genes coding for uncharacterized proteins. Moreover, during the writing of the present manuscript, at least three new *Magallana gigas* genome assemblies were made public in the NCBI repository alone, and one of them (GCF_963853765.1, Wellcome Sanger Institute) has replaced the “reference” status of the genome assembly in this study. None of the currently available genome assemblies derives from a U.S. sourced Pacific oyster. High genetic and molecular diversity in bivalves is thought to be amply rooted in presence/absence variation found through pan-genome studies involving individuals from geographically distant populations [50]. In Pacific oysters, this variation has been found across European populations [51, 52]. In addition, not all current extant genetic variation could be captured with the set of samples analyzed here. Our samples come from a range of geographies and time points in which we acknowledge that, to evaluate extant diversity, sampling more localities at a single time would be the best approach but we made use of samples we had available at the time the study which still capture both spatial and temporal variation. Despite those limitations, this study provides a collection of variants present in Pacific oyster populations and gives insight into the effects of hatchery and artificial crossing.

## Conclusions

In this study, we confirmed that North American Pacific oysters harbor very high genetic heterozygosity. The divergence between Pacific oyster populations along the U.S. Pacific coast is very low but detectable and significant. The captive population in our set, which has been used for breeding for over 25 years, is the most genetically differentiated and shows low nucleotide diversity attributable to the effects of domestication and inbreeding. In addition, this captive population seems to harbor loci with a high probability of having been selected as a result of domestication and artificial selection. Overall, the results presented here are indicative of high-gene flow and weak but detectable population structure among the contemporary populations in this set. The genetic variability detected could be exploitable for breeding purposes and probably confers Pacific oysters with an increased ability to adapt to changing environments.

## Supplementary Information

Additional file 1. Outlier analysis output.

## Data Availability

Sequencing data is available in the NCBI SRA under accession BioProject # PRJNA1165834. Other data is provided within the manuscript or supplementary information.
